# Senile Cardiac Calcification Syndrome: A Rare Case of Extensive Calcification of Left Ventricular Papillary Muscle

**DOI:** 10.4021/cr29w

**Published:** 2011-05-20

**Authors:** Eun Jin Kim, Bong Gun Song, Hyung Rae Sohn, Su-Min Hong, Dong Won Park, Seung Hye Heo, Kye Yeon Kim, Wook-Hyun Cho, Suk-Koo Choi

**Affiliations:** a Division of Cardiology, Cardiac and Vascular Center, Department of Medicine, Inje University Seoul Paik Hospital, Inje University School of Medicine, Seoul, Korea

**Keywords:** Senile cardiac calcification syndrome, Papillary muscle calcification

## Abstract

Extensive papillary muscle calcification is uncommon and only scarce literature about causes and the clinical significance is available, whereas small calcific deposits are common findings in elderly people and are located most commonly at the apex. Papillary muscle calcification has been associated with coronary artery disease, dilated cardiomyopathy, mitral valve disease, hypercalcemia, and increased calcium phosphate product in end stage renal disease. We reported a rare case of extensive calcification of anterolateral papillary muscle diagnosed by echocardiography and multidetector computed tomography.

## Introduction

Papillary muscle calcification has been associated with coronary artery disease, dilated cardiomyopathy, mitral valve disease, hypercalcemia, and increased calcium phosphate product in end stage renal disease [1, 2]. Extensive papillary muscle calcification is rare seen and only scarce literature about causes and the clinical significance is available, whereas small calcific deposits in the apices of the papillary muscles are common findings in elderly people and appear to have no functional consequence [1, 2]. We reported a rare case of extensive calcification of anterolateral papillary muscle diagnosed by echocardiography and multidetector computed tomography.

## Case Report

**Figure 1:**
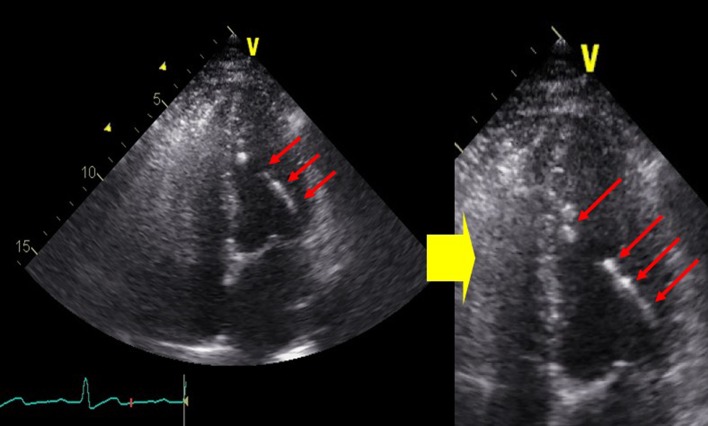
Two dimensional transthoracic echocardiogram showed a spindle-like extensive calcification of anterolateral papillary muscle.

**Figure 2:**
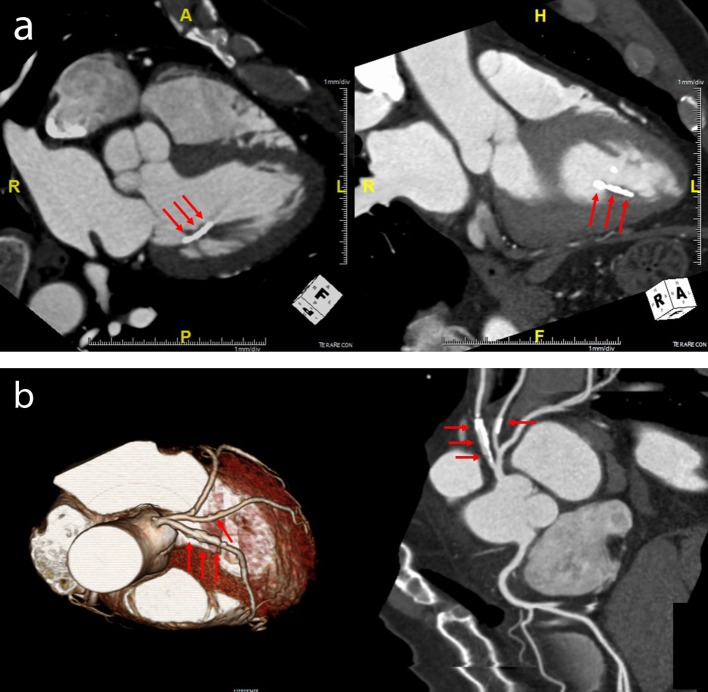
(a) Multidetector computed tomography demonstrated a spindle-like extensive calcification of anterolateral papillary muscle (arrow); (b) diffuse mild stenosis with calcified plaques in proximal portion of left anterior descending coronary artery disease and mild segmental stenosis with calcified plaques in proximal portion of obtuse marginal artery (arrow).

A 67-year-old man with history of hypertension visited our hospital for further evaluation of abnormal findings on two-dimensional transthoracic echocardiogram (TTE) performed during a routine check up examination. He had no past history of any chronic heart disease and chronic renal disease. The patient did not have any symptoms and his physical examination was normal. Initial electrocardiogram and chest roentgenogram revealed no pathology. On TTE, there was a spindle-like extensive calcification in anterolateral papillary muscle (Fig. 1). Left ventricle (LV) had normal chamber size (48 mm at end-diastole and 31 mm at end-systole) and wall dimensions (interventricular septal wall thickness: 7 mm and LV posterior wall thickness: 8mm) and systolic function measured as 64%. Mitral annular and aortic cuspal calcifications were not seen on TTE. TTE did not show significant regurgitations or stenoses of more than mild grade at mitral or aortic valves. His treadmill test showed negative result, which was performed during the routine check up examination. Lipid profiles including low density lipoprotein (LDL)-cholesterol and triglyceride levels were within normal range (69 mg/dl for LDL-cholesterol, 130 mg/dl for triglyceride). A biochemical profile showed normal renal function (blood urea nitrogen: 16.9 mg/dl, creatinine: 0.8 mg/dl) and electrolyte levels including a normal serum calcium (8.7 mg/dl, reference range: 8.4-10.2 mg/dl) and phosphate (3.5 mg/dl, reference range: 2.5 - 4.5 mg/dl). We performed 64-slice multidetector computed tomography, which demonstrated the extensive calcification of anterolateral papillary muscle (Fig. 2a), and diffuse mild stenosis with calcified plaques in proximal portion of left anterior descending coronary artery and mild segmental stenosis with calcified plaques in proximal portion of obtuse marginal artery (Fig. 2b). The patient was managed medically with aspirin and beta blocker and is still free of symptoms 6 months after initial presentation.

## Discussion

Calcium deposits in the heart are common in older persons in areas where symptomatic atherosclerosis is common [1, 2]. Common locations of calcification are atherosclerotic plaques in the epicardial coronary arteries, followed by the mitral annulus, aortic valve cusps, and apices of the left ventricular papillary muscles [1, 2]. In younger individuals, calcific deposits in the epicardial coronary arteries not only indicate the presence of atherosclerotic plaques, but they nearly always indicate the presence of significant luminal narrowing of the arteries containing the calcified plaques. In the elderly aged greater than 65 years, calcific deposits in the epicardial coronary artery do not necessarily indicate the existence of severe luminal narrowing [2, 3]. In the previously published study from necropsy persons aged greater than 65 years, 100% of those with mitral annular calcification had calcific deposits in the aortic valve cusps and in the one or more coronary arteries [4]. The factors that predispose to atherosclerosis in the coronary arteries also predispose to calcific deposits in the mitral annulus and aortic valve cusps [2-4]. Therefore, it has been believed that both mitral annular and aortic cuspal calcific deposits in the elderly have the same pathophysiology as the coronary atherosclerotic plaques [2-4].

Papillary muscle calcification has been described in association with coronary artery disease, dilated cardiomyopathy, mitral valve disease, hypercalcemia, and increased calcium phosphate product in end stage renal disease [5-7]. The posteromedial papillary muscle is dependent on its single blood supply from the right coronary artery, although sometimes also from the left circumflex coronary artery. In contrast, the anterolateral papillary muscle has a dual supply [1, 2]. The papillary muscles have been known to be the last portions of the heart to be perfused with arterial blood, which making the papillary muscle prone to necrosis or sclerosis in the case of an ischemic event [1, 2]. Extensive papillary muscle calcification is rare seen and only scarce literature about causes and the clinical significance is available, whereas small calcific deposits in the apices of the papillary muscles are common findings in elderly people and appear to have no functional consequence [1, 2]. Our case has some striking features. First, extensive papillary muscle calcification is in this case is a rare finding in echocardiographic examinations. Previously published reports have described that calcific deposits of the LV papillary muscles were limited to their apical portions [1, 2, 5-7]. Second, to our knowledge, this is the first report showing extensive calcification of the anterolateral papillary muscle. There are only few cases in the literature of posteromedial papillary muscle calcification after inferoposterior myocardial infarction [1]. Third, extensive papillary muscle calcification in our case is not associated with severe coronary artery narrowing, and therefore, the cause of extensive calcification in this case could not be known because of low possibility of post-ischemic papillary muscle calcification and dysfunction. Also, papillary muscle calcification in this case is not associated with mitral annular or aortic valve cuspal calcifications. We presented a rare case of extensive calcification of anterolateral papillary muscle diagnosed by echocardiography and multidetector computed tomography.
